# Inhibitory effects of crude extracts from some edible Thai plants against replication of hepatitis B virus and human liver cancer cells

**DOI:** 10.1186/1472-6882-12-246

**Published:** 2012-12-06

**Authors:** Wanwisa Waiyaput, Sunchai Payungporn, Jiraphorn Issara-Amphorn, Nattanan T-Thienprasert Panjaworayan

**Affiliations:** 1 Department of Biochemistry, Faculty of Science, Kasetsart University, Bangkok, Thailand; 2 Department of Biochemistry, Faculty of Medicine, Chulalongkorn University, Bangkok, Thailand

**Keywords:** Antiviral activity, Anti-liver cancer, HBV cccDNA, Hepatitis B virus, Edible Thai plants

## Abstract

**Background:**

Edible plants such as *Cratoxylum formosum* (Jack) Dyer, *Curcumin longa* Lin, *Momordica charantia* Lin and *Moringa oleifera* Lam have long been believed in Thai culture to relieve ulcers and the symptoms of liver disease. However, little is known about their anti-liver cancer properties and antiviral activity against hepatitis B virus (HBV). The aim of this study was to investigate the anti-liver cancer and anti-HBV activities of crude extracts from these edible plants on human liver cancer cells.

**Methods:**

Plant samples were prepared and extracted using buffer and hydro-alcoholic solvents. The MTT assay was performed to investigate the effects of the plant extracts on the cell viability of HepG2 cells. The inhibitory effect on replication of HBV was analysed by determining the level of HBV covalently closed circular DNA (cccDNA) in transiently transfected HepG2 cells with the DNA expression plasmid of the HBV genome using a quantitative real-time PCR.

**Results:**

Buffer and hydroalcoholic extracts from *C. formosum* (leaf) reduced cell viability of HepG2 cells and they also inhibited HBV cccDNA. Crude extracts from *C. longa* (bulb) in both solvents did not have any cytotoxic effects on the HepG2 cells, but they significantly decreased the level of HBV cccDNA. Buffer extracts from the leaves of *M. charantia* and the fruits of *M. oleifera* showed to have anti-HBV activity and also a mild cytotoxicity effect on the HepG2 cells. In addition, leaves of *M. Oleifera* extracted by hydroalcoholic solvent drastically decreased the level of cccDNA in transiently transfected HepG2 cells.

**Conclusion:**

Some crude extracts of edible plants contain compounds that demonstrate anti-liver cancer and anti-HBV activities.

## Background

Hepatitis B virus (HBV) infection is a major global health problem that can cause liver cirrhosis and liver cancer. Although the HBV vaccination can effectively prevent new infections, current HBV antiviral drugs are reported to have adverse effects on patients or promote the development of drug-resistance [1]. Therefore, novel antiviral drugs and alternative treatments are urgently needed for millions of people that are chronically infected with HBV. Currently, much attention has been focused on dietary factors that reduce the risk of cancers [2]. Natural products or phytonutrients appear to be potent therapeutics for viral diseases and cancers [3-5].

A number of edible plants in Thailand have been traditionally used to cure illness. For example, the roots and leaves of *Cratoxylum formosum* (Jack) Dyer can be used in a diuretic based diet that may relieve symptoms of liver cirrhosis. Bulbs of *Curcumin longa* Lin has been consumed to treat stomach ulcers and is believed to help prevent liver cancer [6]. The pods and leaves of *Moringa oleifera* Lam have been taken orally to fight cancers and control inflammation. The fruits and leaves of *Momoridica charantin* Linn have been consumed to cure symptoms of liver diseases [7]. In addition, several studies have confirmed the medical properties of these edible plants. For example, curcumin extracted from *C. longa* has been reported to have anti-inflammatory and antioxidant properties and shows antiviral activity for HBV and hepatitis C virus [8-10]. Oil and emulsion extractions from leaves from *C. formosum* have been reported to have high antioxidant activity [11]. 70% methanol extraction of bark from *C. formosum* has been demonstrated to have diuretic effects [12]. Fruit extracts of *M. charantia* have displayed anticancer and antiviral activities against HIV and herpes virus [13,14]. Extracts of *M. oleifera* has shown to inhibit the replication of herpes virus and Epstein-Barr virus [15,16]. Nonetheless, the anti-liver cancer and anti-HBV properties of *C. formosum*, *M. charantia* and *M. oleifera* have not yet been investigated.

This study therefore investigated inhibitory effects of crude extracts from *C. formosum*, *M. charantia* and *M. oleifera* on the viability of human liver cancer cells (HepG2) and their antiviral activity against the replication of the template of HBV transcription (Covalently closed circular DNA or cccDNA). *C. longa* hot water extract was included in this study as the positive control for the anti-HBV property.

## Methods

### Preparation of plant materials

Four edible Thai plants, *C. longa*, *C. formosum*, *M. charantia* and *M. oleifera* were purchased from a local market in Bangkok. The plants were dried under shade for 3 weeks and grounded into powder and stored at 4°C until further use. The powder was then extracted by two different solvent systems, the first being a 80% hydroalcoholic solvent (80% ethanol and 20% distilled water) and the second, a 50 mM Tris-HCl buffer (pH 7.5). For hydroalcoholic extraction, samples were shaken in 80% hydroalcoholic solvent for 16 h at room temperature. Then, the solutions were filtered, and then the filtrate was re-suspended and shaken in ethanol for 48 h. All extracts were pooled, re-filtered and concentrated using a rotary evaporator and then lyophilized. Prior to testing, the lyophilized extracts were dissolved in distilled water to produce 1 g/L of plant extracts. The extract yield (%, w/w) was determined from all hydroalcoholic extracts by using the formula:

(1) $$\text{Yield}\;\left(\%\right)\;=\;\frac{\left(\text{The weight of extract after lyophilisation of solvent}\;\times \;100\right)}{\text{The weight of the plant powder}}$$

(Additional file 1: Table S1).

For buffer extraction, the samples were incubated in buffer for 48 h at 4°C in the dark, filtered, and then centrifuged at high speed for 30 min at 4°C. The supernatant of each sample was collected and dialysed in Tris-HCl for 48 h, then total protein was determined using the Bradford assay, using BSA as a protein standard. The total protein of each buffer extract is presented in Additional file 1: Table S1.

### Cell culture and transfection

Both COS-7 and HepG2 (ATCC, gifts from The Centre of Excellence Clinical Virology and Molecular Biology Research, Chulalongkorn University) were cultured in DMEM (Gibco, Invitrogen) and supplemented with 10% (v/v) heat inactivated fetal bovine serum (Gibco, Invitrogen) and 1%(v/v) antimicotic antibiotic (Gibco, Invitrogen). All cell lines were cultured in 75 cm^3^ sterile tissue culture flasks at 37°C under a 5% CO_2_ atmosphere. For quantitative real time-PCR, HepG2 cells were seeded on 24-well plates with approximately 1 x 10^5^ cells in each well. Seeded cells were treated with either 30 μg/mL of hydroalcoholic crude extracts or buffer extracts containing 0.3 μg total protein and then transfected with 1 μg of the DNA expression plasmid of HBV genome (pHBV48, a gift from M-H Lin, National Taiwan University) [17] and using the Lipofectamine™ 2000 in triplicate. This plasmid has been reported to express full-length HBV and is able to produce HBV particles in cells [17]. For the positive control, cells were transfected with 1 μg of pHBV48 without addition of crude extracts. The ratio between Lipofectamine™ 2000 (μL) and DNA (μg) was 3:1. After 5 days of incubation, cells were subjects to DNA extraction and real-time analysis.

### Cell viability assay (MTT assay)

COS-7 and HepG2 cells were seeded on 96-well plates with a cell density of approximately 1.5x 10^3^ cells per 150 μL of media in each well. After 24 h of incubation, cells were added with 0 (solvent only), 50, 150 and 300 μg/mL of hydroalcoholic extracts. For buffer extracts, cells were incubated with 0 (buffer only), 0.5, 1, 1.5 and 2 μg of total protein extracts from each plant. A cell-free control was also included to exclude any false positive results from the assay [18]. Each sample was performed in quadruplicate and cells were incubated for 3, 5 and 7 days. After incubation, the culture medium was removed from each well by aspiration and 4 μg of MTT (Invitrogen) was added into each well. After 3 h of incubation, DMSO (Amresco) was added to dissolve the purple formazan of MTT. The absorbance was then measured by a microplate reader at a wavelength of 570 nm. The cell viability (%) was calculated using the formula:

(2) $$\frac{\left(\text{Absorbance of test compound}\;-\;\text{Absorbance of cell free control}\right)}{\left(\text{Absorbance of cell without treatment}\;-\;\text{Absorbance of cell free control}\right)}\;\times \;100$$

### Quantitative real time- PCR analysis of HBV cccDNA

Total DNAs were isolated from each well using a genomic DNA Extraction mini kit (RBC Bioscience) according to the manufacturer’s instruction. HBV cccDNA was amplified and quantified by real-time PCR assay using specific primers: HBV_CCC_F1 (5^′^- actcttggactccagcaatg-3^′^) and HBV_CCC_R1 (5^′^-ctttatacgggtcaatgtcca-3^′^) with SYBR-green. These pair of primers was designed to match the region corresponding to the gap and incomplete region in the partially double- stranded HBV DNA. Therefore, they specifically amplify DNA fragments from HBV cccDNA but not from genomic DNA [19]. Negative control (no DNA template) was included to determine contamination whereas the positive control (transfected cells with 1 μg of pHBV48) was performed to yield quantitative information. Each sample was performed in triplicate. A result was indicated in terms of a relative quantitation by the comparative threshold (delta-delta Ct) method, (2^-ΔΔCt^). In this study, the reference gene was beta-globin. The target gene was the cccDNA of transfected HBV and the calibrator was cells transfected with only the HBV plasmid.

### Statistical analysis

Student’s t-test [20] was used for comparing data between control cells (COS-7) and human liver cancer (HepG2) cells in the viability assay. It also used to determine significant differences between the positive control (transfected cells with pHBV48 without addition of crude extracts) and treated cells with crude extracts in the real-time analysis. A statistic t was calculated using the formula:

(3) $$\begin{array}{ccc} \text{t}=\;\frac{\left({\bar{\text{X}}}_{1}-{\bar{\text{X}}}_{2}\right)}{\sqrt[{S_{p}}]{\frac{1}{{\text{n}}_{1}}\;+\;\frac{1}{{\text{n}}_{2}}}} & \text{when} & S_{p}^{2}\;=\;\frac{\left[\left({\text{n}}_{1}-1\right){\text{S}}_{1}^{2}\;+\;\left({\text{n}}_{2}\;-\;1\right)S_{2}^{2}\right]}{{\text{n}}_{1}\;+\;{\text{n}}_{2}\;-2} \end{array}$$

$\bar{\text{X}}$ and ${\bar{\text{X}}}_{2}$ are means of % cell viability of COS-7 cells and HepG2 cells respectively; S_p_ is the sample standard deviation (uncertainty value); S_1_^2^ is COS-7 sample variance; S_2_^2^ is HepG2 sample variance; n_1_ is number of COS-7 sample and n_2_ is number of HepG2 sample. The t distribution was used with the degree of freedom (df) = n_1_ + n_2_ -2. A p-value was determined from the probability table [20]. A P value < 0.05 indicated the presence of a statistically significant difference.

## Results and discussion

### Effects of crude extracts on the viability of HepG2 cells

**Figure 1:**
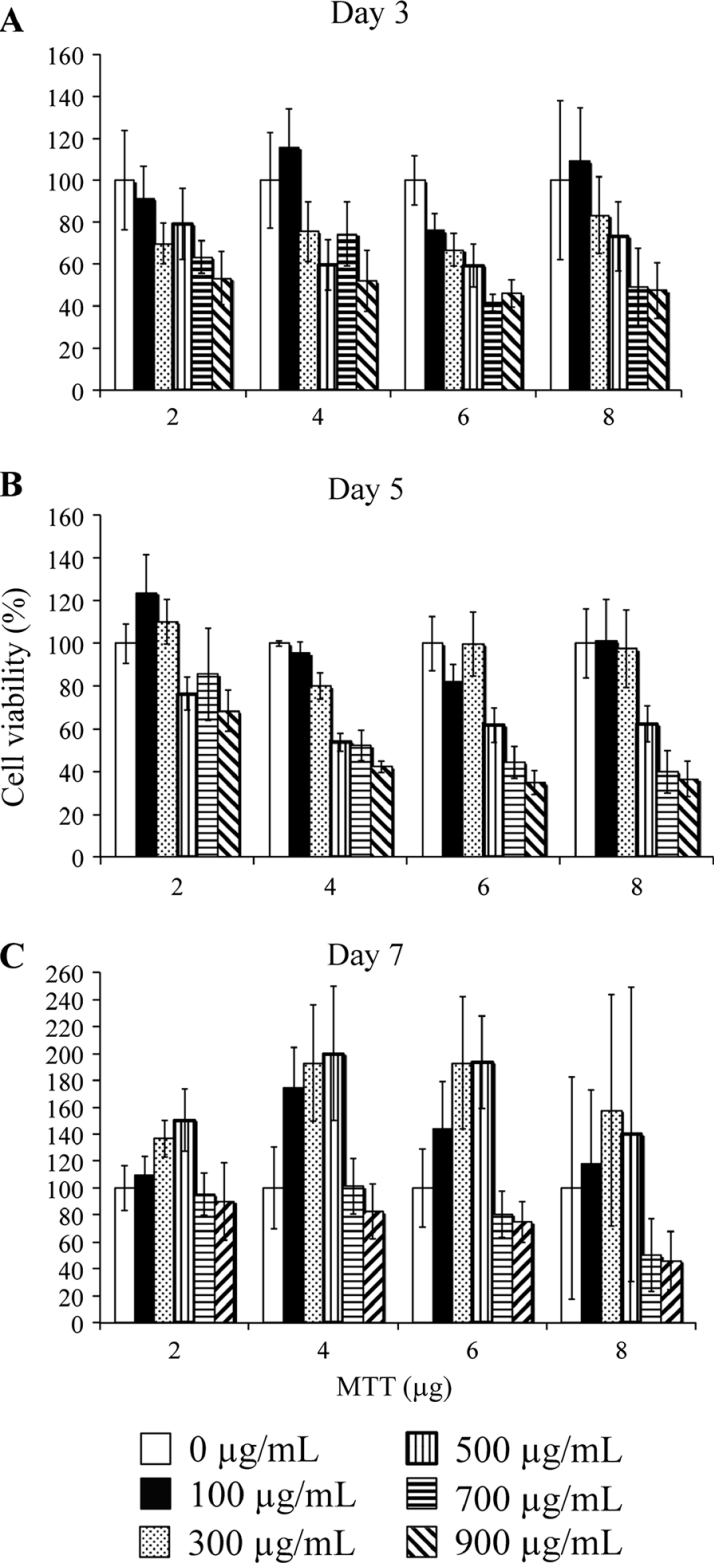
**Optimisation experiment of MTT assay on COS-7 cells.** Cells were treated with various concentrations of *C. longa* hot water extract as indicated in quadruplicate. After 3 days (**A**); 5 days (**B**); and 7 days (**C**) of incubation, cells were subjected to viability analysis using MTT assay with different amounts of MTT (2-8 μg). Bar graph represents a mean value whereas an error bar indicates the uncertainty value of three independent experiments.

**Figure 2:**
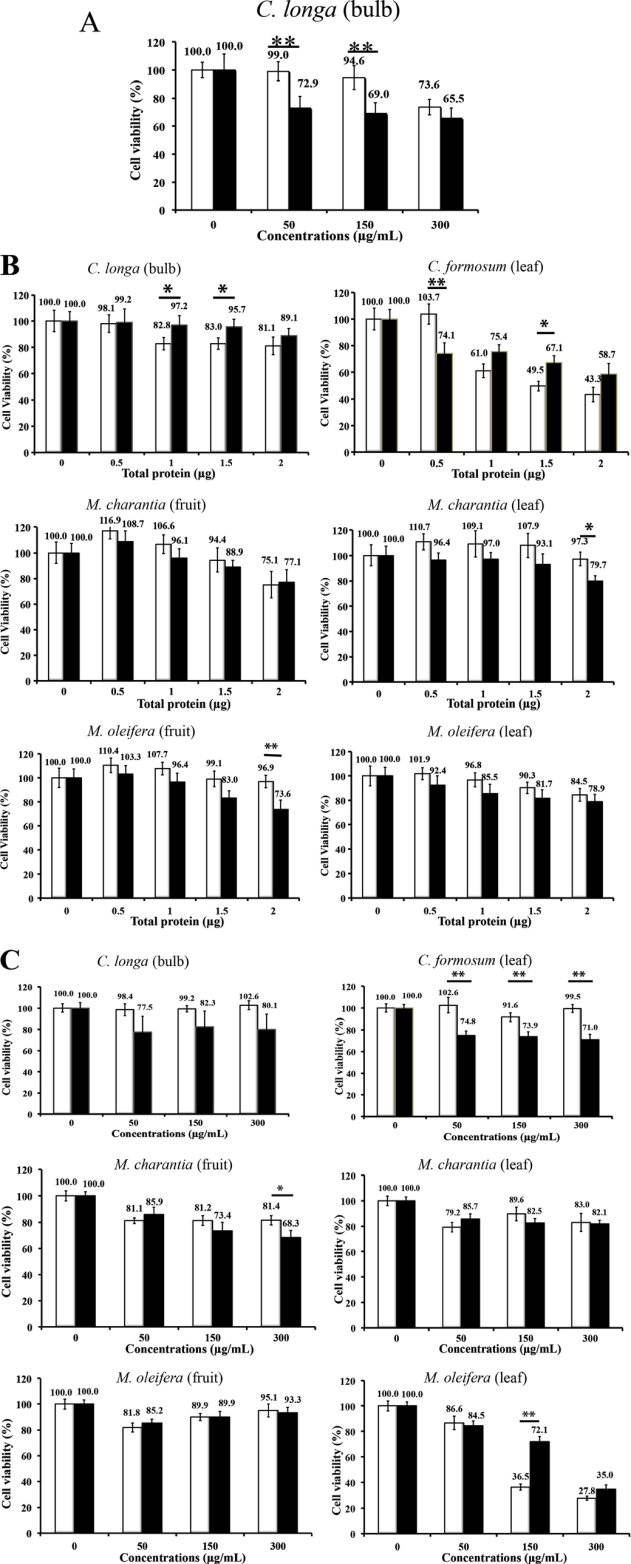
**The cytotoxicity effects from plant extracts on COS-7 and HepG2 cells**. (**A**) The effect from hot water extract of *C. longa* (bulb), (**B**) The effects from buffer extracts and (**C**) The effects from 80% hydroalcoholic extracts. The white bars represent the percentage of cell viability of COS-7 cells whereas the black bars represent the percentage of cell viability of HepG2 cells. Bar graph represents a mean value whereas an error bar indicates the uncertainty value of three independent experiments. “*” and “**” indicated significant inhibitory effect when compared with both cell lines at p < 0.01 and p < 0.001 (by t-test) respectively.

To examine whether extracts from tested edible Thai plants could inhibit the viability of liver cancer cells, an optimisation of the MTT assay was performed on the non-cancerous cell (COS-7) for evaluating: (i) the appropriate concentration of crude extract needed such that there was no cytotoxic effects on the normal cells, (ii) the amount of MTT compound needed and (iii) the duration of effects. We used hot water extracts of *C. longa* as our analytical control because its cytotoxicity and anti-HBV activity were previously reported on the liver-cancer cells [9]. Subsequently, COS-7 cells were treated with various concentrations of *C. longa* hot water extract (100-900 μg/mL) and different amounts of MTT (2-8 μg) and then the MTT assay was performed after 3, 5 and 7 days post-incubation. A high variation of cell viability was observed when cells were treated with 8 μg of MTT while using 2 μg of MTT produced an unclear pattern of dose dependent effects at any time setting (Figure 1). At day 5, the cytotoxicity effect of *C. longa* extract was moderately elevated in a dose dependent manner with 4 μg of MTT and the concentrations of extracts at higher than 300 μg/mL drastically affected cell viability (less than 65% of cell viability, Figure 1B). At day 7, the inhibitory effect was absent and most of the cells were found to be unhealthy when viewed under a microscope leading to relatively high variation of data (Figure 1C). Therefore, the appropriate amount of MTT was 4 μg and the optimal concentration of *C. longa* hot water extract that had no effect on the normal cells was less than 300 μg/mL. The best inhibitory effect was observed at day 5. These optimal conditions were used when analysing the inhibitory effects of all crude extracts on COS-7 and HepG2 cells. The data analysed at day 5 were shown as bar graphs in Figure 2 (see supplemental information for the actual numbers of percentage of cell viability, Additional file 1: Table S2).

From Figure 2A, our data indicated that 50 and 150 μg/mL of hot water extracts from the bulbs of *C. longa* could specifically affect cell viability of HepG2. However, Kim *et al*. (2009) reported that 500 μg/mL of *C. longa* hot water extract had no cytotoxicity effect on the same kind of cell. This variation of concentration may be due to the different conditions used for the MTT assay. Interestingly, crude extract of *C. longa* by Tris-HCl buffer did not inhibit cell viability on HepG2 cells (Figure 2B). This result may suggest that proteins are not involved in the inhibitory effects. Nonetheless, higher amount of total protein may be needed to confirm the MTT result of *C. longa* buffer extract. Curcumin is the product obtained by solvent extraction of *C. longa* and is known to have anti-liver cancer activity [21]. However, our result showed that cytotoxicity effect of 80% hydro-alcoholic *C. longa* crude extract on HepG2 cells had no significant differences from the COS-7 cells (Figure 2C). Therefore, the higher concentration of *C. longa* 80% ethanol extraction may be required to have an effect on the cells.

For the buffer extracts, the results indicated that 0.5 μg of total protein extraction from leaves of *C*. *formosum* significantly decreased cell viability of the HepG2 cells without affecting cell viability of COS-7 cells (Figure 2B: p < 0.001). A similar observation was found for 2 μg of total protein extraction from the leaves of *M*. *charantia* and the fruits of *M. oleifera* (Figure 2B: p < 0.01 and 0.001 respectively). However, higher amounts of protein extracts from the leaves of *C. formosum* seemed to have a greater cytotoxic effect on normal cells than the HepG2 cells. Up to now, there has not been a report on biological active peptides or hepatoprotective proteins from the extraction of *C. formosum.* Therefore, it is very interesting to examine novel peptides or a proteomic profile of *C. formosum* buffer extract. In addition, the crude extract of *C. formosum* leaves by 80% ethanol displayed a significant difference of inhibitory effect between the HepG2 and COS-7 cells (Figure 2C: p < 0.001). This effect is likely due to chlorogenic acid, dicaffeoylquinic acid and ferulic acid derivatives that are previously reported to play important roles in the anticancer activity of *C. formosum*[11]. Notably, crude extracts by 80% ethanol from the fruits and leaves of *M. charantia* and *M. oleifera* did not significantly inhibit cell viability of HepG2 cells (Figure 2C). Therefore, the MTT results (Figure 2) suggested that the crude extracts of *C. formosum* are shown to have the best cytotoxicity effect against liver cancer cells whereas hydroalcoholic extracts of *M. oleifera* (fruit and leaf) and *M. charantia* (fruit and leaf) do not contain compounds that act specifically against the anti-liver cancer activity.

### Some crude extracts of edible plants that could significantly decrease the level of HBV cccDNA in HepG2 cells

**Figure 3:**
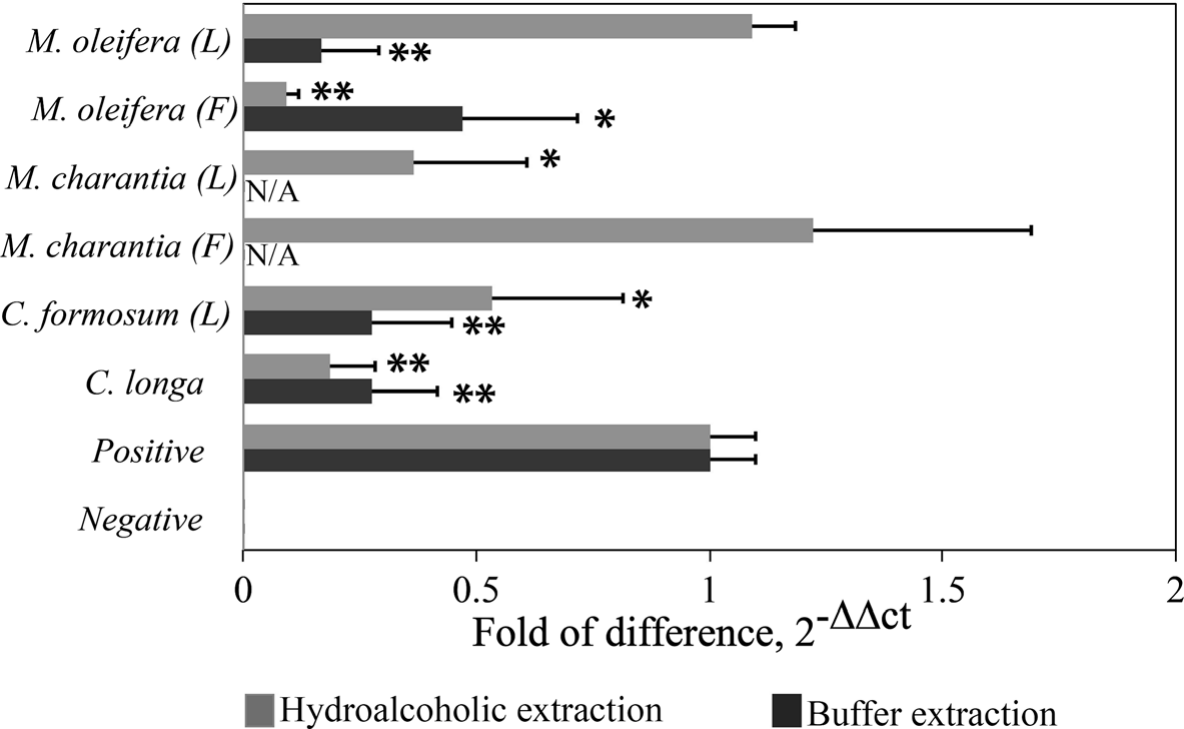
**Effects of crude extracts on the level of cccDNA in transiently transfected HepG2 cells with the DNA expression plasmid of HBV genome.** HepG2 cells were treated with either 30 μg/mL of hydroalcoholic extracts or 0.3 μg of buffer extracts and were transfected with 1 μg of the DNA expression plasmid of HBV genome using Lipofectamine ™ 2000 in triplicate. After 5 days of incubation, the total DNA of each well was extracted and subjected for quantitative real-time analysis. Bar graph represents a mean value whereas an error bar indicates the uncertainty value of three independent experiments. “*” and “**” indicate significant inhibitory effect when compared with the positive cccDNA at p < 0.01 and p < 0.001 (by t- test) respectively. “F” is for fruit and “L” is for leaf.

To study the effect of crude extracts on the replication of HBV, the level of HBV transcription template (cccDNA) was determined using quantitative real-time PCR. This experiment included *C. longa* hot water extract as the analytical control and we found that the extract significantly inhibited cccDNA of HBV with 85% inhibition (data not shown). This result was consistent with the previous report indicating that *C. longa* hot water extract has anti-HBV activity [9]. In addition, almost all the buffer extracts (0.3 μg of total protein) significantly decreased the level of HBV cccDNA in transiently transfected HepG2 cells with the DNA expression plasmid of HBV genome (Figure 3). The best inhibition from the buffer extractions was observed in *C. longa* (bulb), *C. formosum* (leaf) and *M.oleifera* (leaf) with about 80% inhibition whereas *M. oleifera* (fruit) showed to have a moderate inhibitory effect with about 50% inhibition. Notably, buffer extracts from the fruits and leaves of *M. Charantia* were found to drastically reduce gene expression of the reference gene (beta-globin) when compared with untreated cells, thus we did not analyse their effects on the level of HBV cccDNA (Figure 3). This is the first report on the anti-HBV activity of crude extracts by the buffer method from *C. longa* (bulb), *C. formosum* (leaf) and *M.oleifera* (leaf). Further experiments will be carried out to investigate any potential anti-HBV compounds from these plant extracts including their mechanism inside the cells.

Furthermore, our results suggest that 30 μg/mL aqueous ethanol extracts of *M. olifera* (fruit) and *C. longa* (bulb) drastically decreased the level of HBV cccDNA with greater than 85% inhibition. The 30 μg/mL hydroalcoholic extracts of *C. formosum* (leaf) and *M. Charantia* (leaf) also showed a moderate inhibition of the level of HBV cccDNA with 50% inhibition. Hence, our results suggested that crude extracts from 80% ethanol of *C. longa* (bulb), *C. formosum* (leaf), *M. olifera* (fruit) and *M. Charantia* (leaf) contain compounds that could inhibit HBV replication. On the other hand, 30 μg/mL ethanol extracts of *M. Charantia* (fruit) and *M. oleifera* (leaf) had no effect on the level of HBV cccDNA (Figure 3). Therefore, the *M. oleifera* (leaf) ethanol extracts have antiviral activities against herpes simplex virus type 1 (HSV-1) [15] and Sindbis virus [22], but not HBV.

## Conclusion

In this study, we provided the first report on the cytotoxicity effect against liver cancer cell and anti-HBV activity from crude extracts of *C. formosum* (leaf) by using 80% ethanol and Tris-HCl solvents. A mild cytotoxicity effect on HepG2 cells was also observed with the buffer extracts of *M. charatia* (leaf) and *M. oleifera* (fruit), and the hydroalcoholic extracts of these plants were also able to inhibit HBV cccDNA. These results are promising and are to be followed with further analysis of isolated phytochemicals and proteins from these extracts including the identification of the molecular processes that may be targeted by components in these plant extracts.

## Competing interests

The authors declare that they have no competing interests.

## Authors’ contributions

WW carried out the sample preparation and extraction, some MTT assays and real-time PCR. SP designed and was involved in the analysis of real-time PCR experiments. JI carried out protein dialysis and total protein assay. NTP performed some MTT assays, conceived the idea, designed and coordinated the study and prepared the manuscript. All authors read and approved the final manuscript.

## Pre-publication history

The pre-publication history for this paper can be accessed here:

http://www.biomedcentral.com/1472-6882/12/246/prepub

## Supplementary Material

Additional file 1 **Table S1.** The extract yield (%, w/w) of hydroalcoholic extracts and total protein of buffer extracts. Table S2. Effects of crude extracts on the cell viability of COS-7 and HepG2 cells. Click here for file
