# Supplementary material for: Inhibitory effects of crude extracts from some edible Thai plants against replication of hepatitis B virus and human liver cancer cells
Source: BMC Complement Altern Med. 2012 Dec 6;12:246. doi: 10.1186/1472-6882-12-246 (PMC3553072; doi:10.1186/1472-6882-12-246)
Supplement: Additional file 1 — Table S1. The extract yield (%, w/w) of hydroalcoholic extracts and total protein of buffer extracts. Table S2. Effects of crude extracts on the cell viability of COS-7 and HepG2 cells. [file 1472-6882-12-246-S1.doc]

# Supplemental information

Table 1S: The extract yield (%, w/w) of hydroalcoholic extracts and total protein of buffer extracts

| **Edible Plants** | **Part used** | **Yield (%)** | **Total protein (μg/ mL)** |
| --- | --- | --- | --- |
| *Curcuma longa* Linn. | Bulb | 34.42 | 188.54 |
| *Cratoxylum formosum* | Leaf | 28.28 | 222.20 |
| *Momordica charantia* Linn. | Fruit  Leaf | 28.81  35.00 | 53.01  70.83 |
| *Moringa oleifera* Lam. | Fruit  Leaf | 30.00  31.32 | 81.94  160.76 |

**Table 2S: Effects of crude extracts on the cell viability of COS-7 and HepG2 cells.** Cells were treated with various concentrations/amounts of crude extracts as indicated in quadruplicate. After 5 days of incubation, cells were lysed and cell lysates were analysed for cell viability using the MTT assay. For each sample, mean of percentage of cell viability and uncertainty value from three independent experiments were indicated. “*” and “**”indicate significant inhibitory effect when compared HepG2 cells with the COS-7 cells at p < 0.01 and p < 0.001 (by t- test) respectively. ND is not determined

| **Extraction Method** | **Quantity/ Concentration** | **Plant species (Tissue) / Viability effect (%)** | | | | | | | | | | | |
| --- | --- | --- | --- | --- | --- | --- | --- | --- | --- | --- | --- | --- | --- |
|  |  | *C. longa*  (bulb) | | *C. formosum*. (leaf) | | *M. charantia* (fruit) | | *M. charantia*. (leaf) | | *M. oleifera* (fruit) | | *M. oleifera* (leaf) | |
|  |  | COS-7 | HepG2 | COS-7 | HepG2 | COS-7 | HepG2 | COS-7 | HepG2 | COS-7 | HepG2 | COS-7 | HepG2 |
| Hot distilled water  (Control) | 50 µg /mL | 98.99 ± 6.79 | 72.86 ± 8.26****** | ND | | ND | | ND | | ND | | ND | |
|  | 150 µg /mL | 94.60 ± 8.55 | 68.96 ± 7.95****** |  |  |  |  |  |  |  |  |  |  |
|  | 300 µg /mL | 73.57 ± 5.45 | 65.50 ± 7.50 |  |  |  |  |  |  |  |  |  |  |
| 50 mM Tris-HCl buffer  (pH 7.5) | 0.5 µg | 98.13 ± 6.66 | 99.23 ± 9.97 | 103.74 ± 7.54 | 74.09 ± 8.03* | 116.84 ± 6.21 | 108.69 ± 8.42 | 110.72 ± 6.25 | 96.37 ± 5.68 | 110.39 ± 6.03 | 103.30 ± 6.94 | 101.91 ± 4.92 | 92.41 ± 7.7.9 |
|  | 1 µg | 82.79 ± 4.70 | 97.25 ± 6.95***** | 60.97 ± 8.11 | 75.41 ± 5.39 | 106.57 ± 7.19 | 96.15 ± 6.67 | 109.06 ± 10.23 | 97.03 ± 5.28 | 107.73 ± 5.40 | 96.37 ±5.28 | 96.76 ± 5.75 | 85.48 ± 7.72 |
|  | 1.5 µg | 82.96 ± 4.43 | 95.71 ± 5.85***** | 49.50 ± 3.67 | 67.11 ± 5.41* | 94.39 ± 9.41 | 88.89 ± 5.29 | 107.90 ± 9.50 | 93.07 ± 8.34 | 99.09 ± 6.56 | 83.00 ± 6.10 | 90.27 ± 4.58 | 81.68 ± 6.98 |
|  | 2 µg | 81.13 ± 6.71 | 89.11 ± 5.38 | 43.27 ± 5.37 | 58.75 ± 7.89 | 75.12 ± 10.30 | 77.07 ± 9.97 | 97.26 ± 5.40 | 79.70 ± 4.34* | 96.92 ± 5.21 | 73.60 ± 7.96** | 84.54 ± 5.30 | 78.88 ± 6.15 |
| 80% Hydro-alcoholic solvent | 50 µg /mL | 98.35 ± 5.49 | 77.52 ± 2.63 | 102.63 ± 7.20 | 74.83 ± 4.16****** | 81.08 ± 2.23 | 85.89 ± 5.38 | 79.20 ±3.76 | 85.68 ± 3.98 | 81.85 ± 3.56 | 85.17 ± 3.20 | 86.64 ± 5.30 | 84.50 ± 3.63 |
|  | 150 µg /mL | 99.21 ± 2.97 | 82.30 ± 2.61 | 91.60 ± 4.06 | 73.90 ± 4.30****** | 81.21 ± 3.78 | 73.39 ± 6.50 | 89.63 ± 5.36 | 82.48 ± 3.44 | 89.89 ± 2.76 | 89.92 ± 4.40 | 36.50 ± 2.27 | 72.14 ± 3.92****** |
|  | 300 µg /mL | 102.63 ± 4.16 | 80.13 ± 3.23 | 99.49 ± 3.72 | 71.00 ± 4.84****** | 83.04 ±7.16 | 68.32 ± 5.53***** | 83.04 ± 7.16 | 82.07 ± 2.54 | 95.10 ± 5.17 | 93.33 ±4.00 | 27.83 ± 1.61 | 34.96 ± 3.22 |
